# The attitudes and treatment practices of Hungarian primary care dentists regarding dental care for patients with haemophilia

**DOI:** 10.1038/s41598-025-11818-w

**Published:** 2025-07-19

**Authors:** Kitti Sipos, Ildikó Márton, Marianna Móré, Attila Csaba Nagy, Csongor Kiss

**Affiliations:** 1 Department of Operative Dentistry and Endodontics, Faculty of Dentistry, University of Debrecen, Debrecen, 4032 Hungary; 2 Department of Biochemistry and Molecular Biology, Faculty of Medicine, University of Debrecen, Debrecen, 4032 Hungary; 3 Institute of Social and Sociological Sciences, Faculty of Health Sciences, University of Debrecen, Nyíregyháza, 4400 Hungary; 4 Department of Health Informatics, Faculty of Health Sciences, University of Debrecen, Debrecen, 4028 Hungary; 5 Division of Pediatric Hematology and Oncology, Department of Pediatrics, Faculty of Medicine, University of Debrecen, Debrecen, 4032 Hungary

**Keywords:** Haemophilia A, Haemophilia B, Practice patterns, Dentists, Primary health care, Surveys and questionnaires, Dental public health, Special care dentistry, Epidemiology

## Abstract

Patients with haemophilia (PWH) frequently encounter difficulties in accessing dental services, yet research into dental professionals’ attitudes is limited. The present study was thus undertaken to investigate the attitudes and treatment practices of Hungarian dentists in the dental management of PWH. A cross-sectional survey was conducted between September 2022 and December 2022 among dentists working in the primary care sector. An anonymous self-administered questionnaire was utilized for data collection. Pearson’s Chi-squared test and Fisher’s exact test were used for statistical analysis. Variables with statistical significance (*p* < 0.05) were included in multiple logistic regression analyses. A total of 162 dentists consented to participate in the study. Most respondents (*n* = 113, 70%) reported a lack of both confidence and information about treating PWH. The study found that male gender (OR: 3.43, 95% CI: 1.49–7.90), experience in treating PWH (OR: 4.06, 95% CI: 1.73–9.52) and additional qualifications (PhD or DSc) beyond a basic dental degree (DMD/DDS) (OR: 9.40, 95% CI: 1.10-80.48) significantly influenced confidence. Dentists with a minimum of five years of experience demonstrated a greater degree of caution when performing procedures in a primary dental care setting when compared to their younger counterparts. Dental practitioners should receive support to manage PWH confidently.

**Supplementary Information:**

The online version contains supplementary material available at 10.1038/s41598-025-11818-w.

## Introduction

Haemophilia is a rare, X-linked congenital bleeding disorder, characterised by a deficiency of coagulation factor VIII (FVIII), known as haemophilia A, or factor IX (FIX), known as haemophilia B. These factor deficiencies are the result of pathogenic variants in the F8 and F9 clotting factor genes^1^. Haemophilia is predominantly manifested in males, while female individuals are typically heterozygous carriers of a mutated gene^2^.

Haemophilia is characterised by spontaneous bleeding episodes that most often occur in muscles (haematomas) or joints (haemarthrosis). The severity of the disease is categorised based on the level of residual FVIII or FIX activity in the plasma: severe (< 1 international unit (IU)/dl), moderate (1–5 IU/dl) or mild (5–40 IU/dl)^1^. Spontaneous bleeding is common in severe haemophilia, whereas in moderate or mild haemophilia, bleeding is most usually initiated by a provoking factor such as injury, surgery or tooth extraction. Carriers usually show no symptoms^3^.

Both FVIII and FIX deficiency result in reduced or absent activity of the Xase complex preventing the formation of activated factor X (FXa). The manifestation of haemophilia A and B is virtually indistinguishable on the basis of symptoms^4^.

In the context of dental considerations, observation of an unexpected bleeding episode following dental procedure, even more commonly following oral injury or in course of tooth eruption in children, may attract attention for the presence of inherited bleeding disorders (IBD). The role of dentists in the recognition of these conditions is of utmost relevance^1,5–7^.

It has been reported in anecdotally that elderly individuals with haemophilia who underwent dental extractions during childhood had experienced formation of substantial, friable clots in the dental socket in the absence of treatment. Moreover, heavy bleeding returned after a temporary cessation following tooth extraction (“re-bleeding”)^3^. This phenomenon can be attributed to the fact that patients with haemophilia (PWH) exhibit relatively normal initiation and amplification phases of coagulation. Consequently, these patients are able to form an initial platelet plug at the site of bleeding; however, they are unable to generate a sufficient thrombin burst at the platelet surface that is necessary to stabilise the initial platelet plug into a firm fibrin clot^8,9^.

Haemophilia is one of the most prevalent IBD, yet these patients represent a minority of the general population^10^. Consequently, dentists may not acquire confident knowledge and treatment practice^11,12^. The risk of blood-borne infections resulting from earlier plasma-derived factor concentrates decreased the eagerness of dentists to provide care for PWH^13^. Nevertheless, effective oral hygiene practices and regular dental examinations are of paramount importance in preventing dental diseases, a fact that assumes even greater significance in the context of the haemophilia patient population^7,14^. Prolonged bleeding following dental treatment in PWH has the potential to result in serious, and occasionally life-threatening complications. Consequently, it is advised that invasive procedures should be avoided wherever feasible. It is imperative that dentists are aware of the level of risk associated with each procedure, in order to minimise the possibility of complications arising from bleeding^15,16^. Moreover, establishment of an effective professional contact between dentists and haematologists are supposed to facilitate the planning of preventive measures and to discuss optimal haemostatic management. This collaborative approach is crucial in ensuring the minimization of complications in patients.

Previous studies, including our recently published survey, reported that PWH had difficulties accessing dental services^11,17–22^. Most studies focused on the patients’ perspective, except of a single report from the UK incorporating the views of dentists, too^11^.

The objective of this study was to examine the attitudes and treatment practices of Hungarian dentists in treating PWH. We focused on primary care, both government funded and private practices, as the majority of patients do not require advanced level care and can be treated in general dental practice (GDP) by adhering to established protocols. Furthermore, dentists working in primary care are the first and most appropriate point of contact for patients within the current Hungarian health insurance system.

## Methods

### Study design

This national cross-sectional study was conducted between September and December 2022 among dentists working in the primary care sector in Hungary. The selection criteria were specifically designed to include only dentists working at primary care level in Hungary. This category includes dentists working in GDPs registered with the National Health Insurance Fund of Hungary and private practices. The present study excluded dentists who exclusively worked at a higher level of care (i.e. in a specialist institution or care centre); those who participated in the pilot study to develop the survey questionnaire; and dentists who did not answer the question on informed consent.

Data were collected via an anonymous self-administered questionnaire based on literature data and expert opinion provided by the senior dentist and a haematologist co-authors (IM and CK)^11,12^. A pilot test of the questionnaire was conducted with a sample of 30 dentists employed at the University of Debrecen, Faculty of Dentistry, who were responsible for providing advanced level care. Ethical approval was obtained (Regional and Institutional Ethics Committee, Clinical Centre, University of Debrecen; No. DE RKEB/IKEB: 6087 − 2022). Study was conducted in compliance with the Helsinki Declaration.

### Sample population and data collection

Participants were selected from a list of GDPs from the website of the National Health Insurance Fund of Hungary in September 2022. A priori sample size calculation was performed with a power level of 80% and an ɑ level of 0.05 using proportions (25.81% and 49.07%)^23^.

Selected dentists were invited to participate in the survey via email. All participants provided informed consent by affirmative response given to the first question of the questionnaire. Participation was voluntary.

Of the 163 randomly selected dentists 47 returned the questionnaire (response rate: 28%). In consideration of the low rate of participation, an invitation to contribute was extended to private practice dentists (*n* = 115) who were affiliated with the University of Debrecen, specifically those engaged in the practical graduate dental student training programs and/or regularly participating in the postgraduate training programs organized by the University of Debrecen. Questionnaires were sent to dentists who had replied positively to the invitation.

A total of 162 dentists provided consent by completing the questionnaire, which was either submitted online via a Microsoft Forms link (*n* = 114; 70%) or returned by post (*n* = 48; 30%).

### Variable specifications

The questionnaire contained 21 questions and was divided into four domains (Table S1).

The statistical analysis considered four academic titles, i.e. Doctor of Dental Medicine degree (DMD), Doctor of Dental Surgery degree (DDS), Doctor of Philosophy degree (PhD), and Doctor of Science of the Hungarian Academy of Sciences degree (DSc). According to the Hungarian Specialist Training Program, three groups of professional experience were defined (< 2 years: Resident Doctor; 2–5 years: Specialist Candidate and Young Specialist; >5 years: Senior Specialist). This study investigated three major regions of Hungary (Great Plains and North Hungary; Central Hungary (the capital city of Budapest + Pest county) and Transdanubia).

### Variable selection and statistical analysis

Categorical variables were compared using Pearson’s Chi-squared test and Fisher’s exact test. Multiple logistic regression model was built to investigate the factors influencing the dentists’ self-reported confidence in providing dental care to PWH. Findings from the logistic regression analyses were represented as odds ratios (ORs) and 95% confidence intervals (CIs). Statistical evaluations were executed using STATA IC Version 18.0 software^24^. A p-value < 0.05 was considered significant.

## Results

### Characteristics of the dentists participating in the research

Of the 162 participants, 97 (60%) were female and 65 (40%) were male. Mean age was 36.22 years (Standard Deviation (SD) = 10.34) (see Table 1 for more details).

**Table 1:** Overview of dentists participating in the research.

Characteristic	%(*n* = 162)
**Gender** Female Male	59.88 *(97)* 40.12 *(65)*
**Title/Qualification** DMD/DDS DMD + PhD DMD + PHD + Dsc	92.59/2.47 *(150/4)* 4.32 *(7)* 0.62 *(1)*
**Professional experience in years** < 2 2–5 > 5	9.26 *(15)* 20.99 *(34)* 69.75 *(113)*
**Specialist certificate** No specialization One specialization More than one specialization	41.36 *(67)* 44.44 *(72)* 14.20 *(23)*
**Group of patients served** Children Adults Both	2.47 *(4)* 51.85 *(84)* 45.68 *(74)*

### Haemophilia care from a dental perspective

The majority (*n* = 113; 70%) of surveyed dentists did not consider themselves confident and properly informed in treating PWH in primary practices (Table 2).

One hundred and one/162 (62%) respondents never treated PWH. Ninety-eight/101 (97%) never encountered such a patient. Three/101 (3%) did not provide care for PWH because they did not feel informed regarding their proper care.

Dentists with no experience of treating PWH exhibited significantly lower confidence levels (82.18%), whereas confidence judgement was balanced among those with PWH treatment experience (50.82% confident vs. 49.18% not confident, *p* < 0.001; Table 2). Gender was also a significant factor, with men being more confident (*p* = 0.001; Table 2). Years of professional experience and specializations did not have a significant impact on confidence, but scientific qualifications obtained beyond the DMD/DDS degree (PhD/DSc) did (*p* = 0.005; Table 2). No regional differences were found (Table 2). Majority of respondents (140/162; 86.42%) expressed that management of PWH should be emphasized in undergraduate curricula and postgraduate training. Most respondents (85%) would participate in such training courses. Confidence was significantly influenced by the questions about postgraduate training and the willingness to participate (Table 2; *p* = 0.008, *p* = 0.036, respectively).

**Table 2:** Dentists’ self-reported confidence in providing dental care to PWH.

Variables	Not confident *n* = 113	Confident *n* = 49	*p*-value (Pearson’s Chi-squared test)
Not treated PWH	82.18%	17.82%	< 0.001*
Treated PWH	49.18%	50.82%	
Male	55.38%	44.62%	0.001*
Female	79.38%	20.62%	
Professional experience in years			
< 2	66.67%	33.33%	0.906
2–5	67.65%	32.35%	
> 5	70.80%	29.20%	
Title/Qualification			
DMD/DDS	72.08%	27.92%	0.005*
DMD/DDS + other (PhD/DSc)	25.00%	75.00%	
Specialist certificate			
No specialization	70.15%	29.85%	0.996
One specialization	69.44%	30.56%	
More than one specialization	69.57%	30.43%	
Region of employment (Hungary)			
Great Plains and North Hungary	61.33%	38.67%	0.082
Central Hungary	80.00%	20.00%	
Transdanubia	74.47%	25.53%	
Greater emphasis in undergraduate curricula on bleeding conditions and their dental treatment options			
No	59.09%	40.91%	0.242
Yes	71.43%	28.57%	
The need for further training on postgraduate courses in the dental care of patients with bleeding disorders			
No	45.45%	54.55%	0.008*
Yes	73.57%	26.43%	
Participation in a training course on the dental care of patients with bleeding disorders			
No	52.00%	48.00%	0.036*
Yes	72.99%	27.01%	

﻿A multiple logistic regression model was developed to examine the effect of factors on self-reported confidence in providing dental care to PWH. The model identified three significant factors among investigated variables used in pearson’s Chi-squared test analysis. Male dentists (OR: 3.43, 95% CI: 1.49–7.90), respondents with treatment experience (OR: 4.06, 95% CI: 1.73–9.52), and those with additional qualifications beyond basic dental degrees (OR: 9.40, 95% CI: 1.10-80.48) were more likely to report confidence in providing dental care to PWH (Table 3).

**Table 3:** Multiple logistic regression analysis of the factors associated with self-reported confidence in providing dental care to PWH.

Variable	Evaluation of the factors that contribute to self-reported confidence in haemophilia dental care	
	OR (95% CI)	*p*-value
Gender (Male/Female)	3.43 [1.49–7.90]	0.004*
Experience in treating PWH (Yes/No)	4.06 [1.73–9.52]	0.001*
Additional qualifications (Yes/No)	9.40 [1.10-80.48]	0.041*

**Fig. 1:**
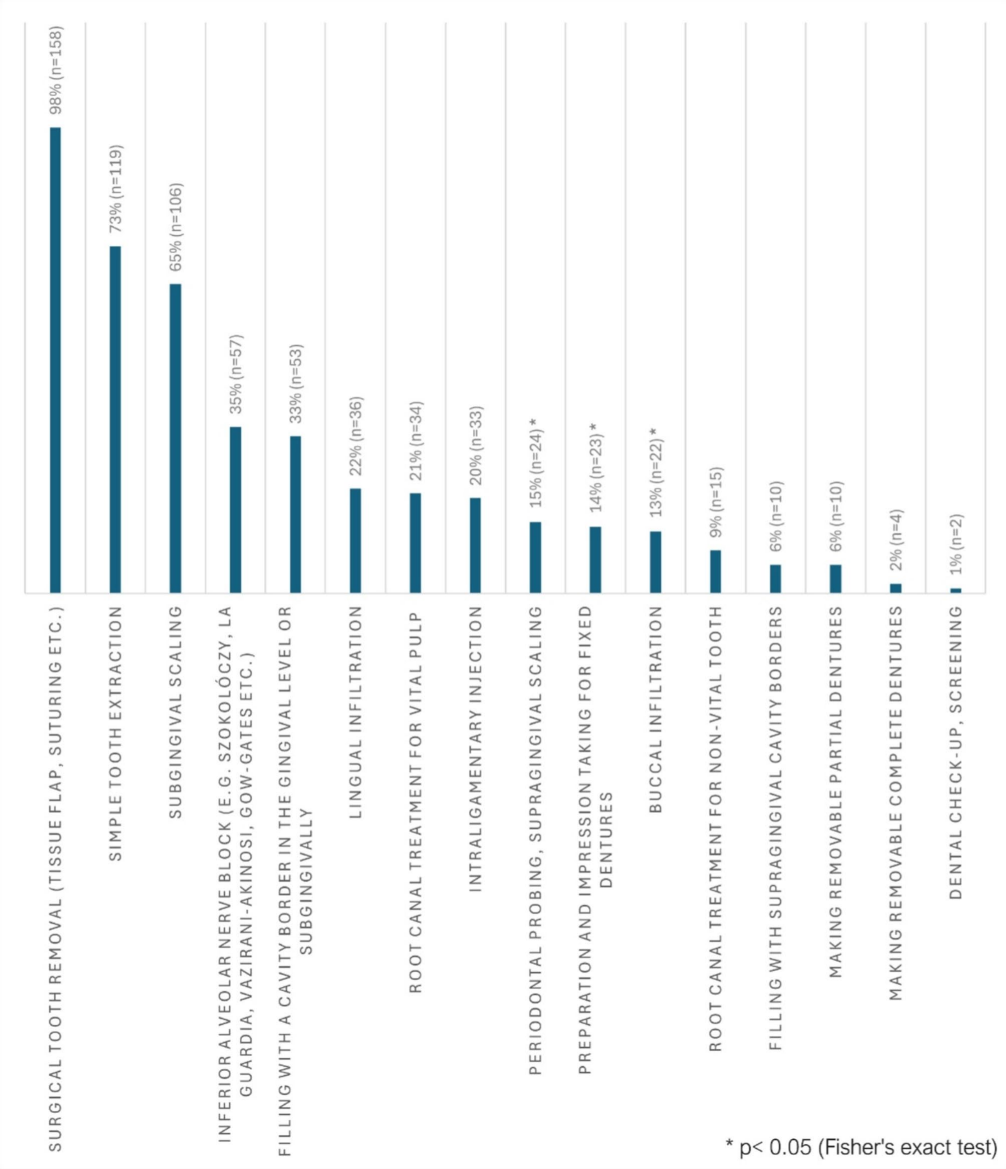
Dental procedures referred by dentists working in primary care to an advanced level of care (multiple choices).

In response to the question selecting dental procedures of PWH to be referred to advanced level care, the most frequently mentioned procedures were surgical tooth removal (*n* = 158; 98%), simple tooth extraction (*n* = 119; 73%), and subgingival scaling (*n* = 106; 65%) (Fig. 1).

Fisher’s exact test was employed to examine the correlation between the years of professional experience and the referral to advanced dental care. A total of 100% of those with 2–5 years of work experience would perform periodontal probing and supragingival scaling (*n* = 34; *p* = 0.005), as well as buccal infiltration technique (*n* = 34; *p* = 0.008) and would not refer PWH to advanced care facility (Table 4).

The findings indicated a tendency towards a higher rate of referrals for each intervention among those practicing for over five years (*n* = 113, 70%), when compared to younger colleagues (< 2 years, and 2–5 years professional experience groups, *n* = 49, 30%). Among various interventions, the context of the preparation and impression taking for fixed dentures was identified as a significant factor, with 19% of respondents in practice for > 5 years indicating that they would refer their patients for higher-level care (*p* = 0.040; Table 4).

**Table 4:** The relationship between years of professional experience and referral intention for periodontal probing and supragingival scaling, buccal infiltration anaesthesia, and preparation and impression taking for fixed dentures.

	Professional experience in years			*p*-value (Fisher’s exact test)	Total
	< 2	2–5	> 5		
Referral for periodontal probing and supragingival scaling procedures					
No	73.33% (*n* = 11)	100% (*n* = 34)	82.30% (*n* = 93)	0.005*	85.19% (*n* = 138)
Yes	26.67% (*n* = 4)	0% (*n* = 0)	17.70% (*n* = 20)		14.81% (*n* = 24)
Referral for buccal infiltration anaesthesia					
No	80% (*n* = 12)	100% (*n* = 34)	83.19% (*n* = 94)	0.008*	86.42% (*n* = 140)
Yes	20% (*n* = 3)	0% (*n* = 0)	16.81% (*n* = 19)		13.58% (*n* = 22)
Referral for preparation and impression taking for fixed dentures					
No	93.33% (*n* = 14)	97.06% (*n* = 33)	81.42% (*n* = 92)	0.040*	85.80% (*n* = 139)
Yes	6.67% (*n* = 1)	2.94% (*n* = 1)	18.58% (*n* = 21)		14.20% (*n* = 23)

Prior to treating PWH, most dentists would request a consultation, particularly with the managing haematologist. Table 5 illustrates the consultation patterns of dentists.

One objective of this survey was to investigate medication choices of dentists. Details pertaining to pain management and antibiotic use can be found in Table 6.

**Table 5:** Consultation patterns of dentists in the dental care of PWH.

Consultation	% (*n* = 162)
Yes (any treatment)	61.11 *(99)*
In case of uncertainty	38.88 *(63)*
**Contact** *(multiple choices)*	
Patient’s haematologist	85.80 *(139)*
Senior dentist/oral surgeon	39.51 *(64)*
Patient’s general practitioner	35.19 *(57)*
Colleagues	20.37 *(33)*

**Table 6:** Prescribing practices of dentists for painkillers and antibiotics.

PAINKILLERS (multiple choices)	% (*n* = 162)
Paracetamol	59.26 *(96)*
Pyrazolone derivatives (phenylbutazone, aminophenazone, dipyrone/metamizole)	32.72 *(53)*
Non-selective Non-Steroidal Anti-Inflammatory Drug (NSAID)	14.29 *(23)*
Selective cyclooxygenase-2 (COX-2) inhibitors (e.g. celecoxib, nimesulide)	11.73 *(19)*
Acetylsalicylic acid	0 *(0)*
I do not know	24.69 *(40)*

Antibiotics *(one answer choice)*	
Amoxicillin	22.22 *(36)*
Clindamycin	1.85 *(3)*
Both of these I would be prescribed	58.64 *(95)*
Neither of these I would be prescribed	0 *(0)*
I do not know	17.28 *(28)*

Most dentists would prescribe a paracetamol painkiller to PWH (*n* = 96; 59%). Amoxicillin was the preferred antibiotic for 22% of respondents, while 2% indicated a preference for clindamycin. The option “I would prescribe both” was selected by the greatest number of respondents (*n* = 95; 59%).

**Fig. 2:**
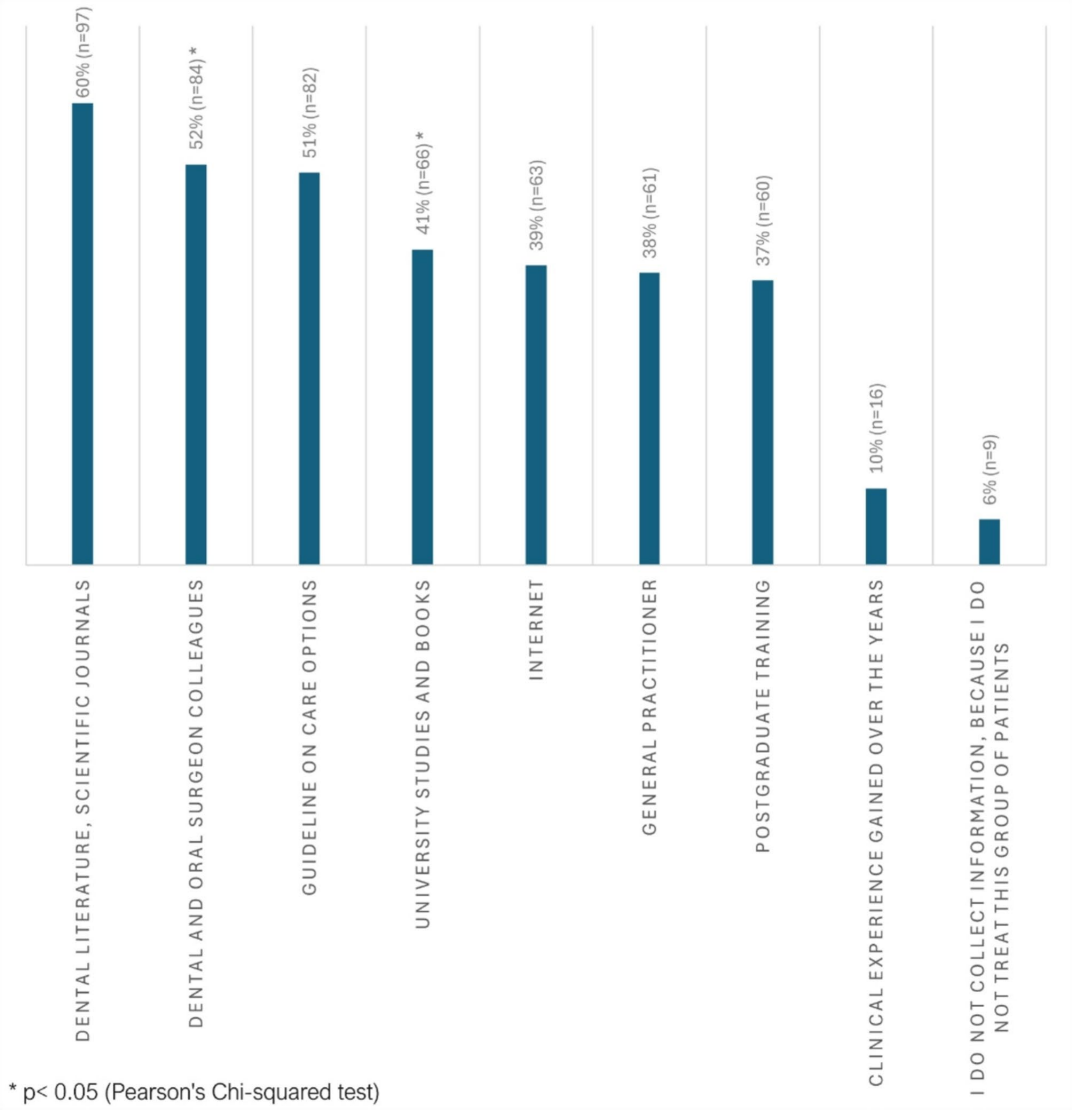
The orientation sources of dentists with regard to the dental care of the PWH (multiple choices).

The survey investigated which sources of information had oriented dentists in providing dental care to PWH. Most dentists (*n* = 97; 60%) use scientific journals as a source of information (Fig. 2).

Correlation between years of professional experience and orientation sources of dentists was examined by Pearson’s Chi-squared test. We found a statistically significant correlation between professional experience and the utilization of previous undergraduate studies and books (*p* < 0.001; Table 7). Professionals with > 5 years of experience were significantly less likely to rely on these resources, whereas those with < 2 years of experience were more likely to utilize them. Moreover, a statistically significant correlation exists between professional experience and the probability of obtaining assistance from dental and oral surgery colleagues. Specifically, professionals with < 2 years of experience were more likely to seek assistance, whereas those with > 5 years of experience were less likely to accept help (*p* = 0.015; Table 7).

**Table 7:** The relationship between years of professional experience and the responses of “I rely on my previous university studies and books” and “I ask my dental and oral surgeon colleagues for help”.

	Professional experience in years			*p*-value (Pearson’s Chi-squared test)	Total
	< 2	2–5	> 5		
Undergraduate studies and books					
No	26.67% (*n* = 4)	41.18% (*n* = 14)	69.03% (*n* = 78)	< 0.001*	59.26% (*n* = 96)
Yes	73.33% (*n* = 11)	58.82% (*n* = 20)	30.97% (*n* = 35)		40.74% (*n* = 66)
Help from dental and oral surgeon colleagues					
No	13.33% (*n* = 2)	47.06% (*n* = 16)	53.10% (*n* = 60)	0.015*	48.15% (*n* = 78)
Yes	86.67% (*n* = 13)	52.94% (*n* = 18)	46.90% (*n* = 53)		51.85% (*n* = 84)

## Discussion

Recently, we have published self-reported experiences of Hungarian PWH regarding access to and experiences with dental treatment. A considerable proportion of patients indicated that, despite the awareness of this condition among dental practitioners, their knowledge was significantly constrained^22^. Here we collected 162 dentists from all regions of Hungary who completed a questionnaire investigating their self-reported opinion on this topic, representing the first study from continental Europe. The research focused on dentists working in primary care, as authors believe that identifying challenges in dentistry should start at this stage of care provision.

Unfortunately, 70% of the surveyed dentists indicated that they felt non-confident lacking the required knowledge to treat PWH. The proportion is considerably higher than that reported in a single similar investigation from the UK, where 40% of dentists expressed a lack of confidence in treating patients with IBD in a GDP setting^11^. Haemophilia is a rare disease, dentists infrequently or never treat PWH, resulting in a deficit of confidence regarding both knowledge and treatment practices^11,12^. In the present survey, 3% of healthcare professionals indicated that they do not treat PWH because of a lack of sufficient information about the most appropriate course of treatment. The relatively low rate is likely attributable to the fact that Hungarian dentists have access to comprehensive Hungarian-language guidance covering all aspects of dentistry and dental care for PWH^25,26^. We feel important that a comprehensive guide should be easily accessible to dentists in their native languages. Particularly that a significant number of respondents indicated that they relied on scientific journals and guidelines as their primary source of information.

It is also recommended that greater emphasis be placed on the care of patients with bleeding disorders in undergraduate dental education. The findings of our study indicated that professionals with < 2 years of experience were more likely to rely on undergraduate studies and books when caring for PWH. In contrast, the majority of those with > 5 years of experience did not utilize these resources. We strongly believe in the importance of providing comprehensive education in the management of PWH at undergraduate level. This ensures that future dental practitioners are well prepared from the outset of their careers. In addition, targeted postgraduate training and continuing education programmes are crucial for senior colleagues to stay informed of new developments. Encouragingly, our findings revealed that many respondents expressed interest in pursuing postgraduate training on this topic, highlighting the need for educational initiatives in this area. The study revealed that participants who obtained additional scientific qualifications (PhD and DSc) in addition to a dental degree, were nine times more likely to report confidence in providing dental care to PWH.

The results of our survey demonstrated that 61% of respondents would pursue a consultation for any dental treatment for PWH, particularly with the managing haematologist. However, a study conducted in the UK revealed a gap in the knowledge of haematologists and nurses regarding dental procedures associated with an increased risk of bleeding^12^. Interdisciplinary team may provide most appropriate dental care to PWH. Professionals with < 2 years of experience were more likely to request or accept help with the dental care of PWH than their senior colleagues. It might be beneficial to have a readily accessible advanced level of point of contact for giving advice and guidance for medical practitioners who do not have sufficient experience with PWH. Even selection of proper painkillers and antibiotics may prove a difficult decision without sufficient practice with PWH.

This study indicated a correlation between prior experience in treating PWH and higher levels of self-reported confidence in providing dental care. The authors consider availability to consultation important since survey results indicated that patients perceived greater safety in dental offices with a hospital background or in dental practices that are linked to haemophilia treatment centres^17,21^. Readily accessible consultation services would enhance access of patients to primary care instead of higher-level care.

Findings of this study revealed a surprising gender gap: male dentists exhibited greater confidence in treating PWH than females. Exact causes of this difference are not known, but review of the literature suggested that men tended to report higher levels of confidence than women in a range of clinical tasks^27,28^. The results obtained from this study have the potential to provide a valuable foundation for future research in this area.

Most dental practitioners in the UK study demonstrated an understanding of the dental treatments that could be provided safely in GDP and those that should be referred to an advanced level of care^11^. In the present study, an examination was conducted of the dental procedures that dentists would typically refer for advanced care, as opposed to those performed in a primary dental care setting (Fig. 1). A significantly higher proportion of dentists with two to five years of experience would not refer their PWH for more advanced levels of care for specific procedures, in comparison to their younger and senior colleagues. This might be due to the fact that they gained more experience than the group with < 2 years of experience. Furthermore, the younger generation may demonstrate a greater propensity for professional development training programmes, with the objective of enhancing their career progression. Consequently, they may possess a more contemporary perspective than some of their senior colleagues. Dentists with > 5 years of experience demonstrated greater caution in their approach to every intervention, particularly, in the case of tooth preparation and impression taking for fixed restorations, in comparison to their younger counterparts.

The principal strength of this study is that it is a nationwide survey, which is particularly important given that it focuses on a less researched area. However, the relatively small sample size may limit the generalizability of the results. Future studies with larger and more diverse samples are needed to extend these findings. There are, nevertheless, several other limitations to this study. GDPs were selected at random from a list published on the official website of the National Health Insurance Fund. However, personal invitation of dentists working in private practices, due to low response rate might biased the results. Furthermore, the findings of this study are limited by the self-reported confidence of the participants, without the objective evaluation of their skills and abilities to provide care. Finally, it should be noted that the absence of open text domains in the questionnaire may have limited the participants’ ability to express their opinion comprehensively.

## Conclusions

The perspective of dental professionals on the treatment of PWH is currently under-researched. Most respondents of this Hungarian survey lack confidence and information about dental care for PWH, highlighting the need for support and assistance for dentists. Most participants had no prior experience with dental management of PWH. The presented data suggest that treating this cohort of patients, dentists became more confident regarding their abilities to provide dental care. It is therefore of the utmost importance that primary care dentists be involved in the dental care of PWH. Such an approach would markedly enhance the standard of dental care that patients receive and mitigate the challenges they currently encounter. Conclusions of this survey would merit from a larger, multi-national Central-East European-based questionnaire dedicated to the investigated issue.

## Electronic supplementary material

Below is the link to the electronic supplementary material.

Supplementary Material 1

## Data Availability

The data that support the findings of this study are available from the corresponding author upon reasonable request due to privacy/ethical restrictions.
