# Supplementary material for: The attitudes and treatment practices of Hungarian primary care dentists regarding dental care for patients with haemophilia
Source: Sci Rep. 2025 Jul 19;15:26261. doi: 10.1038/s41598-025-11818-w (PMC12276225; doi:10.1038/s41598-025-11818-w)
Supplement: Supplementary file 1 — Supplementary Material 1 [file 41598_2025_11818_MOESM1_ESM.pdf]

The attitudes and treatment practices of Hungarian primary care dentists regarding dental care for patients with haemophilia

Kitti Sipos[1], Ildikó Márton[2,3], Marianna Móré[4], Attila Csaba Nagy[5], Csongor Kiss[6]

Supporting information

Supplementary Table 1. Survey questions

1. **Gender of the patient**	
2. **Year of birth**	
3. **What is/are your title(s) upon graduation from the Dental/Stomatology Faculty/School?** (e.g. DMD, DDS, PhD, MDS etc.)	
4. **What year did you start working?**	
5. **Do you have a specialization?** (Mark one answer)	Yes, I have one specialization, which is: …..
	Yes, I have more than one specialization, which are: ……….
	I have no specialization
6. **At what level of care do you work?** (Multiple answers can be marked)	General Dental Office
	Private Practice
	Specialist Institution or Care Centre
7. **City of your dental office/workplace**	
8. **Which group of patients are you mainly involved in caring for?** (Mark one answer)	Children
	Adults
	Both
9. **On average, how many patients do you treat per week?**	
10. **Have you ever treated a patient with haemophilia in your practice?**	Yes
	No
11. **If you answered "no" to question 10, what is the reason?** (Mark one answer)	I have never met a patient with haemophilia in my practice

	I do not treat patients with haemophilia because I do not feel informed about their care
	I do not treat patients with haemophilia because their care involves extra anxiety and strain
	I do not treat patients with haemophilia because I have had negative experiences with their treatment in the past
12. **What dental procedures would you refer to a specialised institution or care centre if you had a patient with haemophilia?** (Multiple answers can be marked)	Dental check-up, screening
	Periodontal probing, supragingival scaling
	Subgingival scaling
	Buccal infiltration
	Intraligamentary injection
	Inferior alveolar nerve block (e.g. Szokolóczy, La Guardia, Vazirani-Akinosi, Gow-Gates etc.)
	Lingual infiltration
	Simple tooth extraction
	Surgical tooth removal (tissue flap, suturing etc.)
	Filling with supragingival cavity borders
	Filling with a cavity border in the gingival level or subgingivally
	Root canal treatment for vital pulp
	Root canal treatment for non-vital tooth
	Preparation and impression taking for fixed dentures
	Making removable partial dentures
	Making removable complete dentures
13. **Would you feel confident and adequately prepared to treat a patient**	Yes
	No

**with haemophilia in your General Dental Practice/ Private Practice?**	
14. **Which of the following painkillers would you recommend for a patient with haemophilia?** (Multiple answers can be marked)	Paracetamol
	Acetylsalicylic acid
	Non-selective NSAID (Non-Steroidal Anti-Inflammatory Drug)
	Selective COX-2 inhibitors (e.g. celecoxib, nimesulide)
	Pyrazolone derivatives (phenylbutazone, aminophenazone, dipyrone/metamizole)
	I do not know
15. **Which of the most commonly used antibiotics in dentistry would you prescribe for a patient with haemophilia?** (Mark one answer)	Amoxicillin
	Clindamycin
	Both of these I would be prescribed
	Neither of these I would be prescribed
	I do not know
16. **If you had to treat a patient with haemophilia, would you consult someone (e.g. haematologist, general practitioner, oral surgeon, etc.)?** (Mark one answer)	Yes I would, whatever the treatment
	I would only consult when if I were unsure
	I would not consult anyone
17. **If you needed a consultation, who would you contact?** (Multiple answers can be marked)	The patient's GP
	The patient’s haematologist
	I would ask a senior dentist/oral surgeon for help
	I would ask one of my peers
18. **From what forums do you get information about dental care for**	From the Internet
	From dental literature, scientific journals
	I am looking for a guideline on care options
	I was informed during postgraduate training
	I rely on my previous university studies and books

**haemophilia patients?** (Multiple answers can be marked)	I ask my dental and oral surgeon colleagues for help
	I ask a GP for help
	I rely on the clinical experience I have gained over the years
	I do not collect information, because I do not treat this group of patients
19. **Do you think more emphasis should be placed in university education on bleeding conditions and their dental treatment options?**	Yes
	No
20. **Do you think there is a need for further training on postgraduate courses in the dental care of patients with bleeding disorders?**	Yes
	No
21. **Would you participate in a training course on the dental care of patients with bleeding disorders?**	Yes
	No
